# When does word frequency influence written production?

**DOI:** 10.3389/fpsyg.2013.00963

**Published:** 2013-12-24

**Authors:** Cristina Baus, Kristof Strijkers, Albert Costa

**Affiliations:** ^1^Center for Brain and Cognition, Universitat Pompeu FabraBarcelona, Spain; ^2^Laboratoire de Psychology Cognitive, CNRS - Universite Aix-MarseilleMarseille, France; ^3^Institució Catalana de Recerca i Estudis AvançatsBarcelona, Spain

**Keywords:** written word production, typewriting, central and peripheral processes, lexical frequency

## Abstract

The aim of the present study was to explore the central (e.g., lexical processing) and peripheral processes (motor preparation and execution) underlying word production during typewriting. To do so, we tested non-professional typers in a *picture typing task* while continuously recording EEG. Participants were instructed to write (by means of a standard keyboard) the corresponding name for a given picture. The lexical frequency of the words was manipulated: half of the picture names were of high-frequency while the remaining were of low-frequency. Different measures were obtained: (1) first keystroke latency and (2) keystroke latency of the subsequent letters and duration of the word. Moreover, ERPs locked to the onset of the picture presentation were analyzed to explore the temporal course of word frequency in typewriting. The results showed an effect of word frequency for the first keystroke latency but not for the duration of the word or the speed to which letter were typed (interstroke intervals). The electrophysiological results showed the expected ERP frequency effect at posterior sites: amplitudes for low-frequency words were more positive than those for high-frequency words. However, relative to previous evidence in the spoken modality, the frequency effect appeared in a later time-window. These results demonstrate two marked differences in the processing dynamics underpinning typing compared to speaking: First, central processing dynamics between speaking and typing differ already in the manner that words are accessed; second, central processing differences in typing, unlike speaking, do not cascade to peripheral processes involved in response execution.

## Introduction

In the last decades, typewriting (e.g., e-mailing, social networks) has become a fundamental tool for our personal and professional communication in daily life, especially in industrial societies. As a result, thousands of emails are written daily around the world. It is surprising then that despite the increasing relevance of such activity, relatively little is known about the underlying processes of writing in comparison to other means of communication (speaking). With the aim of extending our knowledge about written production, in the present study we explored the involvement of central and peripheral processes during single word (written) production.

Writing a word (as well as verbally producing it) requires the involvement of both central-cognitive and peripheral-motor processes (e.g., Margolin, 1984). *Central processes,* on the one hand, are those core linguistic processes that allow individuals to transform their ideas into the appropriate sequence of letters (or sounds) that compose the intended word. For that to be possible, information flows through different levels of processing: semantic, lexical (orthographic long term memory) and sublexical (orthographic working memory) (e.g., Hillis, 2008; Rapcsak et al., 2009). Indeed, *response latencies,* which are considered to reflect central processing, are sensitive to semantic, lexical, and orthographic manipulations (Bonin and Fayol, 2000, 2002; Bonin and Meot, 2002; Bonin et al., 2002, 2012). The *peripheral processes*, on the other hand, are those processes responsible for the engagement of the specific motor plans needed to execute the letters in the desired output modality (e.g., handwriting, typewriting) (see, Purcell et al., 2011). *Response durations* (and also *interkeystroke intervals* in typewriting) have been taken as reflecting operations at more peripheral levels of processing (e.g., Delattre et al., 2006).

Most of the work devoted to investigate written production (especially from the neuropsychological field; see Rapp and Dufor, 2011, for a review) has concerned two main issues: (1) whether information cascades within the central levels of processing similarly in speech and written production (e.g., Hillis et al., 1999), and (2) the relationship between the central and the peripheral processes underlying written production. Specifically, whether central and peripheral processes are staged and therefore independent from each other, or whether central processes cascade (influence) into peripheral processes. (Purcell et al., 2011; see for spoken production, Kello et al., 2000).

Our research aims to contribute to these two central issues in the field of written production by exploring: (1) the electrophysiological correlates of lexical processing within the central stages of typewriting, which will allow us to compare the temporal dynamics of written production with that in the spoken modality, and (2) whether lexical variables known to affect central stages during written production will also influence peripheral motor processes. Lexical frequency was manipulated as an index of lexical processing. As these two questions are relatively independent from each other, in the following we will first focus on the evidence regarding frequency effects within central-cognitive levels both in the written and the spoken modality, and then we will consider the evidence gathered so far regarding frequency effects at more peripheral-motor levels during writing.

Word frequency effects on response latencies have been extensively reported both in the writing and in the spoken modality. Words of high-frequency are written/named faster and more accurately than those of low-frequency (Bonin and Fayol, 2000, 2002; Caramazza and Costa, 2001; Caramazza et al., 2001; Roelofs, 2001; Bonin et al., 2002; Jescheniak et al., 2003; Navarrete et al., 2006; Kittredge et al., 2008; Strijkers et al., 2010). For those models assuming a lexical origin (Dell, 1986; Caramazza, 1997), the frequency effect has been taken as an index of the speed to which lexical representations are accessed. Indeed, electrophysiological, and intracranial studies have provided evidence of an early effect of frequency (Sahin et al., 2009; Strijkers et al., 2010). Around 200 ms after the picture onset presentation, ERP amplitudes corresponding to low-frequency words become more positive than those corresponding to high-frequency words (P2)^1^. Note however, that this early ERP frequency effect does not exclude the possibility of frequency affecting later levels of processing, such as phonological encoding (Jescheniak and Levelt, 1994; Levelt et al., 1999; Almeida et al., 2007; Strijkers et al., 2010).

To the best of our knowledge, no study has explored the electrophysiological correlates of word frequency in typewriting. However, given that a priori there are no reasons to expect lexical effects to arise at different levels for writing and speaking (Perret and Laganaro, 2012; see also, Perret and Laganaro, 2013), frequency ERP effects are expected to arise in a similar time-window as the one observed in picture naming studies, around 200 ms (Strijkers et al., 2010). Moreover, in order to cover the potential effect of word frequency on later (post-lexical) central stages during written production (Perret and Laganaro, 2012), we also focused on later ERP components (e.g., P300).

Regarding word frequency effects at more peripheral-execution processes (e.g., Sternberg et al., 1978), the evidence is scarcer and the results on the influence of lexical frequency on response durations are quite mixed. Some studies have shown shorter writing durations or faster interkeystroke intervals for high-frequency words than for low-frequency ones (e.g., Gentner et al., 1988), which will favor the idea of cascade processing between central and peripheral levels during typewriting (e.g., Sahel et al., 2008). Others have shown no frequency effect on response durations (e.g., Delattre et al., 2006), supporting a more discrete processing (Logan and Zbrodoff, 1998; Damian and Freeman, 2008; Bonin et al., 2012). Thus, the evidence regarding the influence of central processes onto peripheral processes is far from conclusive. However, it should be noted that the different results come from different writing paradigms, which complicates to make generalizations regarding the stage/cascade nature of the underlying processes of written production. For instance, while frequency effects in response durations have been observed in a writing-to-copy task (e.g., transcribe an article; Gentner et al., 1988), no frequency effect was obtained in a writing-to-dictation task (Delattre et al., 2006). So, it is possible that the different demands imposed by the task vary the sensitivity of the peripheral processes to be influenced by the central ones (Delattre et al., 2006). In the present study we tested the frequency effects on response durations in a picture typewriting task, a task that has revealed frequency effects in the spoken modality^2^. Therefore, if central and peripheral processes are closely interrelated as seems to be the case for spoken production, we predict shorter response durations and interstroke intervals for high compared to low-frequency picture names. In contrast, if written production is an activity where central processing has little or no effect on motor execution, both the response durations and the interstroke intervals should be unaffected by the word frequency manipulation.

In sum, in the present study we explored the temporal course of lexical access in typewriting, which will allow us to compare it with the speaking modality (e.g., Strijkers et al., 2010, 2011, 2013). The temporal precision of the ERPs will help us determine the exact moment/s in which word frequency affects written production. To do so, ERPs locked to the picture onset presentation were explored. Moreover, addressing the effect of word frequency in typewriting response durations will allow us to ask whether variables known to affect central processing activity (e.g., lexical access) will affect also response durations and interstroke intervals.

## Methods

### Participants

Twenty Spanish native speakers (11 women) non-professional typers, from the University Pompeu Fabra took part in the experiment. All of them were right-handed, had normal or corrected-to-normal vision and declared not having neurological or motor problems.

### Materials

Ninety-six black and white drawings were selected from the Snodgrass and Vanderwart (1980) and similar databases (Bates et al., 2003) (see Appendix for the full list of materials). Half of the pictures had high-frequency names and the remaining half had low-frequency names. Frequency values were larger for high-frequency (mean = 40.5, *SD* = 41.7) than for low-frequency words [mean = 4.7, *SD* = 2.7; *t*_(95)_ = 5.8, *p* < 0.001]. Words in both conditions were matched in letter length (HF mean: 6 letters, ranging from 4 to 10 letters long; LF: 5.6 letters, 4 to 9 letters long), neighborhood density and frequency of the first syllable (all *t*s < 1). Importantly, high and low-frequency words were matched in their first letter [e.g., bebé (HF, *baby*), bola (LF, *ball*)]. For all the pictures, name agreement ratings were collected. A new group of 24 participants was asked to provide a name for each picture. Name agreement was at 91% for pictures with high-frequency and 89% for those pictures with low-frequency names (*t* < 1).

### Procedure

Participants were instructed to type by means of a QWERTY standard keyboard the Spanish names of the pictures presented on the screen. The trial structure was as follows: (1) a fixation point appeared on the screen for 500 ms, followed by the picture presentation for fixed time duration of 500 ms. After that time the picture disappeared leaving a blank screen that remained until participants finished typing their response. Pictures were presented for a short and fixed time (500 ms), in order to avoid responses before the appearance of the blank screen. This was important given that as participants were allowed to look at the keyboard, movements would carry ocular artifacts in the ERPs. Nevertheless, as participants were non-professional typers and faster responses than 500 ms were not expected, participants were instructed to start typing their responses as soon as they knew the response (no response latency was below 500 ms; see results section). Moreover, they could see and monitor their responses and were allowed to use the delete key whenever they made an error. When participants were confident with their response, they were instructed to press the Enter key for the next trial to start. The keyboard was located on the participants' laps, at enough distance (~60 cm) from their eyes to avoid abrupt movements of the head when looking at the keyboard. The approximate distance between the participant and the center of the screen was 120 cm. Participants were instructed to be still, not to move their head and look to the keyboard (moving only their eyes) as little as possible to avoid EOG artifacts. Reaction times were obtained for each keystroke. Words misspelled (even if self-corrected), or pictures for which the participant used a name different from the one designated by the experimenter were considered as errors and excluded from the analysis.

#### EEG procedure and analysis

The EEG was continuously recorded and linked-nose referenced from 30 scalp Ag/Cl passive electrodes. Eye movements were monitored by two external electrodes placed horizontally (outer canthus) and vertically (below) to the right eye. Impedances were kept below 5 kΩ. EEG signal was digitalized online with a 500 Hz sampling rate and a band pass filter of 0.1–125 Hz. EEG data was filtered offline through a 0.03 Hz high-pass filter and 20 Hz low-pass filter and vertical and horizontal ocular artefacts were corrected (Gratton et al., 1983).

ERPs time locked to the onset of the stimulus were segmented into 750 ms epochs (−200 to 550 ms) and segments with incorrect responses, containing artefacts (brain activity above or below 100 μ V or a change in amplitude between adjacent segments of more than 200 μ V) or eye blinks were excluded. The 750 ms epochs were then averaged in reference to −200 ms pre-stimulus baseline.

***Data analysis*.** We analyzed participants' behavioral and ERP responses. After excluding a participant with more than 25% of errors (one participant) the final analysis included 19 participants. Six words were also eliminated as having too many errors (more than 35%) relative to the rest of the words (below 25%). Behaviorally, three measures were obtained for correct responses: response latencies, response durations and interstroke intervals. Error rates were also analyzed.

***ERP analysis*.** For the ERPs, we selected three time-windows of interest: the P2 time-window (170–230 ms), the N3 (230–330 ms) and the P3 (330–430 ms). A 2 × 9 × 3 ANOVA was conducted considering Word frequency (high vs. low-frequency), Region of interest and Electrode [LeftAnterior (LA): F7, F3, FC5; Fronto-central (FC): Fz, FC1, FC2; RightAnterior (RA): F8, F4, FC6; Left-central (LC): T3, C3, CP5; Centro-Parietal (Cpar): CP1, Cp2, Pz; Right-central (RC): T4, C4, CP6; Left-parietal (LP): T5, P3, PO1; Right-parietal (RP): T6, P4, PO2; Occipital (O): O1, Oz, O2]. Moreover, the onset latency of the frequency effects was explored. ERPs for high and low-frequency words were compared by running a 2-tailed paired *t*-test at every sampling point (every 2 ms) starting from the picture presentation. The onset of the frequency effect was considered to be the first significant data point of a sequence of consecutive sampling points showing significant differences between high and low-frequency words (*p*-values FDR corrected below 0.05; Benjamini and Hochberg, 1995).

## Results

### Behavioral

#### Response latencies

Typing latencies and error rates were analyzed by means of separate *t*-test for participants and items in which high and low-frequency words were compared. We observed a significant frequency effect for typing latencies, [*t*_1(18)_ = −2.8, *p* < 0.05; *t*_2(88)_ = −1.9, *p* = 0.05]. Participants typed faster those pictures with high-frequency names (HF: 1398) than those with low-frequency names (LF: 1483). Error rate analysis revealed no difference between high and low-frequency words (all *t*s < 1).

#### Response durations and interstroke interval

Response durations, calculated as the difference between the onset of the word (first keystroke) and the offset of the word (last keystroke), tended to be shorter for high-frequency words (916 ms) than for low-frequency ones (958 ms) but this difference did not reach significance [*t*_1(18)_ = 1.5, *p* = 0.1; *t*_2_ < 1]. Moreover, no differences were observed in the mean interstroke interval of high (206 ms) and low-frequency words (203 ms) (*t* < 1).

### ERPs

A 2 × 9 × 3 ANOVA considering Frequency, Region of interest (ROI) and electrode was explored in three time-windows: 170–230, 230–330, and 330–430^3^.

**Figure 1:**
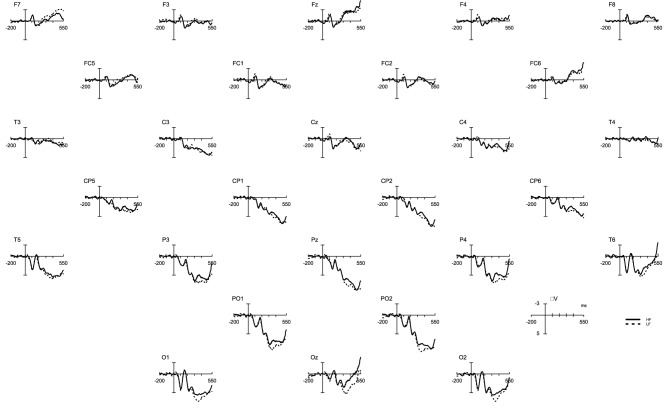
**ERP waveforms for high (solid line) and low-frequency words (dashed line) locked to the onset of the picture presentation**.

**Figure 2:**
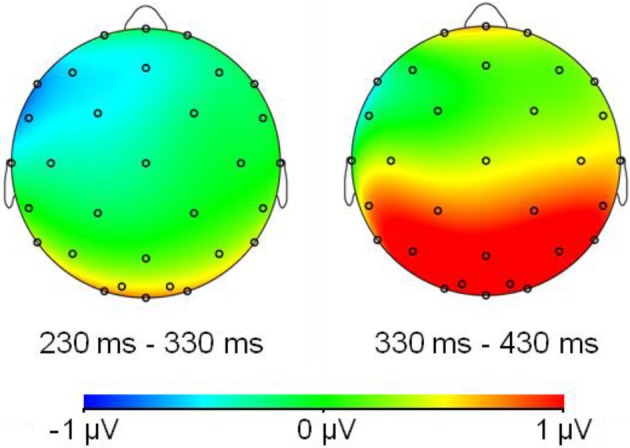
**Topographical maps of the wave difference of low-frequency word amplitudes minus high-frequency word amplitudes**.

Table 1 shows the statistical analyses for each time-window. As indicated, frequency interacted with region at two time-windows. At the time-window between 230 and 330 ms, the frequency effect was only significant for those electrodes located at the LeftAnterior electrode cluster [*F*_(18)_ = 5.01, *p* = 0.03]. In this region, the frequency effect was in the opposite direction as expected; that is, ERP amplitudes for LF words were more negative than those for HF words. In contrast, in the time window between 330 and 430, ERP amplitudes for low-frequency words were significantly more positive than those of high-frequency at the posterior regions [PL: *F*_(18)_ = 4.3, *p* = 0.05; PC: *F*_(18)_ = 6.10, *p* = 0.02; O: *F*_(18)_ = 6.19, *p* = 0.02] (see Figures 1, 2).

**Table 1:** **Statistics resulting from the ANOVAs conducted at each temporal window**.

	**170–230**	**230–330**	**330–430**
Frequency	*F* < 1	*F* < 1	*F*_(1, 18)_ = 3.3, *p* = 0.08
Region	*F*_(1.5, 28.4)_ = 2.1, *p* = 0.14	***F*_(1.7, 31.1)_** = **19.9**, ***p*** = **0.000**	***F*_(1.7, 31.1)_** = **11.3**, ***p*** = **0.000**
Frequency * Region	*F* < 1	***F*_(2.1, 38.9)_** = **3.1**, ***p*** = **0.05**	***F***_**(2, 36.6)**_ = **3.8**, ***p*** = **0.02**
Frequency * Region * Electrode	*F* < 1	*F* < 1	*F*_(3.2, 58.3)_ = 1.7, *p* = 0.17

#### Onset latency analysis

**Figure 3:**
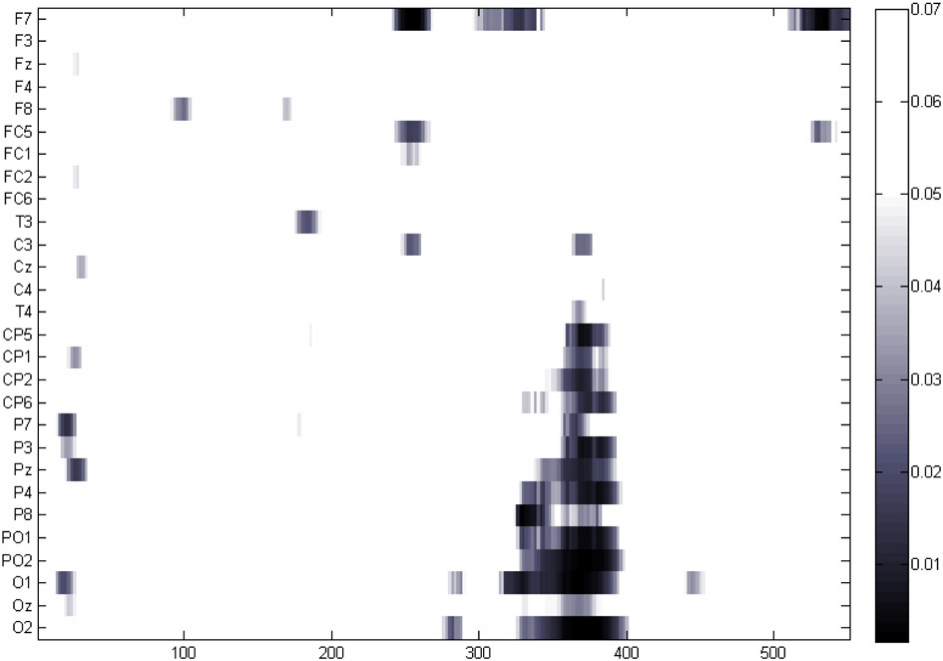
***P*-values resulting from the paired *t*-test (FDR corrected) at each sampling point. Colored points correspond to those *p*-values below 0.05**.

Given the observed ERP frequency effect was maximal over posterior electrodes, onset latency analyses were based only on the mean amplitude of the nine electrodes located at posterior regions (T5, P3, PO1, PO2, P4, T6, O1, Oz, O2). The latency analysis revealed that ERP amplitudes for low and high-frequency words started to diverge significantly at 324 ms after picture onset presentation and the difference remained until 396 ms after the picture onset (*p*-values FDR corrected below 0.05) (see Figure 3).

## Discussion

The aim of the present study was to investigate: (1) the temporal course of lexical access in typewriting, and (2) the discrete/cascade relationship between central and peripheral processes during written word production. By means of a picture typewriting task, we explored the influence of word frequency within the central levels of processing, and whether its influence extends also to peripheral-motor levels of processing. Our results revealed that low-frequency words elicited longer typing latencies and larger amplitudes than high-frequency words. Moreover, the ERP results revealed a frequency effect arising around 350 ms after the picture onset. In contrast, no frequency effect was observed during typing execution: frequency did not influence the duration of the words nor the speed to which the letters composing a given word were typed.

Regarding the influence of lexical frequency within central processes, our results replicate previous findings on the effects of word frequency (e.g., Bonin and Fayol, 2002): High-frequency words are produced faster than low-frequency ones and this occurs regardless of the modality in which the word will be finally produced (written production or speech production). Moreover, low-frequency words elicited more positive amplitudes than those elicited by high-frequency words, replicating the ERP frequency effect observed in the spoken modality (Strijkers et al., 2010, 2011). However, and in contrast to the posterior frequency effect (posterior P2) in the picture naming (e.g., Strijkers et al., 2010), the frequency effect in typing became apparent later in time: high and low-frequency words differed at the time-window between 330 and 430 ms. As we will comment below, the posterior frequency effect was accompanied by an anterior frequency effect with low-frequency words eliciting more negative amplitudes than high-frequency ones.

Why does the posterior lexical frequency effect manifests earlier in speech production than in typewriting? One possibility is that word frequency only affects post-lexical processes in typing while it already modulates initial lexical access in speaking. Indeed, based on the temporal meta-analysis of speech production provided by Indefrey and Levelt (2004), effects after 300 ms are thought to index sublexical processing (phonology/orthography). However, such strategy for assigning levels of representation to time-course is highly tentative (more so since Indefrey and Levelt's estimates are based on an average response latency of only 600 ms) and speculative at best (Strijkers and Costa, 2011). This is especially so when taking into account that it is well documented by now that word frequency is an ubiquitous variable affecting both early and late stages of central processing (e.g., Almeida et al., 2007; Graves et al., 2007; Knobel et al., 2008; Strijkers et al., 2010).

Alternatively, it might be possible that the observed delay in the ERP frequency effects is the result of an overall slow-down of the response latencies (around 1400 ms) relative to the spoken modality (around 700 ms). However, and contrasting this position, other word production studies have shown that the onset of lexical access seems unaffected by the speed of the response. For instance, in three ERP studies of overt naming (Strijkers et al., 2010, 2011, 2013; see also Laganaro et al., 2012) reaction times fluctuated roughly between 600 and 1000 ms, but the onset of the lexical frequency effect remained constant. The latter makes sense since it indicates that the reaction times in naming are influenced by linguistic factors only after initiating lexical selection and not before at pre-linguistic stages of processing, consistent with the predictions of the dominant speech production models (e.g., Dell, 1986; Caramazza, 1997; Levelt et al., 1999). In a similar vein, it seems unlikely to conclude from these findings that picture recognition—the required step prior to engaging the language system—is slower in typing than in speaking. However, what might be different between both production types is the speed with which picture semantics can access words. That is, while we do have daily experience with naming visual information in our environment (e.g., Pass me *that bottle*. Is *that chair* taken? You *look sad*), the same is not true for typing. Therefore, connections from picture semantics with those linguistic representations relevant for speech production (e.g., lexical phonology) could be more strongly linked in the brain than those from picture semantics to representations involved for typing (e.g., orthography), and therefore the latter are accessed in a less automatized and consequently, slower manner. Note that at the core of this assumption lies the idea that the way in which words are accessed from perceived objects is different depending on the type of production task engaged, consistent with the notion that the earliest linguistic modulations are driven (in part) by specific top-down modulations preparing those brain systems and neural pathways relevant to the goal-directed behavior at hand (e.g., Strijkers et al., 2011). Based on the current data we cannot determine whether this explanation is indeed behind the observed pattern, but it certainly involves an excellent question for future research. For now, the important contribution here is the demonstration that the brain's response to word frequency is different between speaking and typing. This finding is at odds with previous claims in the literature that lexical processing is fully shared between the two production modalities (e.g., Perret and Laganaro, 2012, 2013).

To conclude this section, we must comment on the reversed frequency effect at the anterior sites preceding the posterior frequency effect: Between 230 and 330 ms after picture onset low-frequency amplitudes were more negative than high-frequency ones. A similar result was found in a recent study of overt object naming (Strijkers et al., 2013), the only difference between these two studies being the presence of the posterior P2 frequency effect in the latter study (i.e., spoken modality). The authors tentatively proposed this N300 effect could be related to integration of object semantics as encountered in picture processing tasks (e.g., Holcomb and McPherson, 1994; McPherson and Holcomb, 1999; Schendan and Kutas, 2002; West and Holcomb, 2002; Schendan and Maher, 2008). Another possibility is that this negative deflection is associated with cognitive control indexed by the N2 component (Falkenstein et al., 1999; Nieuwenhuis et al., 2003; Folstein and Van Petten, 2008), especially since in language it seems to manifest slightly later in time, peaking around 300 ms after picture onset (e.g., Jackson et al., 2001; Swainson et al., 2003; Christoffels et al., 2007; Verhoef et al., 2009; Martin et al., 2013), with items of low-frequency requiring an increase in cognitive control compared to the high-frequency ones. Nevertheless, whether the current negative anterior ERP effect can be seen as functionally related to those encountered in conceptual processing of object knowledge or recruitment of cognitive control has yet to be established, given that componentry comparisons over very different tasks is difficult (e.g., Picton et al., 2000). However, one may still argue that the language-related frequency effects in the typewriting task are characterized by an anterior negativity rather than by the later posterior positivity. This option seems unlikely, however, given that the anterior effect was very small (it did not survive our correction for multiple comparisons in the onset latency analyses) and localized to only a few scalp electrodes. This indicates a very fine-grained effect generated by a small population of synchronously firing neurons, while a variable as word frequency is known to produce robust and extended ERP modulations in production tasks (e.g., Fiez et al., 1999; Graves et al., 2007; Strijkers et al., 2010, 2011, 2013). With this finding and given the fact that the earliest effects of lexical frequency consistently elicit modulations at posterior sites in both language production and perception (e.g., Hauk and Pulvermüller, 2004; Hauk et al., 2006; Strijkers et al., 2010, 2011, 2013), we maintain that the first modulations of word frequency within the language system are engendered by the posterior positivity between 330 and 430 ms. However, even if we take on the unlikely stance that in typewriting the first language-sensitive modulations of word frequency are reflected in the anterior negativity, that does not compromise our conclusion with respect to the temporal dissociation in lexical access between speaking and writing given that the N300 modulation still occurs about 100 ms later compared to the P2 effects in object naming (more so, it would indicate that not only the time-course but also the neural generators—anterior in case and posterior in the other—are functionally distinct between the two production modalities).

Regarding the second main question we wanted to address in this study, namely whether lexical variables influence the execution of peripheral-motor commands in typewriting, our results did not show frequency effects during response execution, neither in the duration of the word, nor in the speed with which letters were typed. This result is at odds with previous evidence in the spoken and writing modalities showing a negative correlation between the frequency of the word and its articulatory duration (e.g., Gentner et al., 1988; Balota and Chumbley, 1990). In contrast, it favors those proposals assuming that in writing, information flows from central to peripheral processes in a discrete manner (e.g., Damian and Freeman, 2008). That is, central and peripheral processes underlying typewriting are independent from each other. Hence, the influence of lexical variables on the speed with which a word is selected does not extend to the motor commands involved in executing the production of that word^4^. Similarly, the evidence coming from different cases of dysgraphia also supports a clear distinction between central and peripheral processes. Peripheral dysgraphias, are characterized for instance by the repetition, omission, and substitution of letters while writing, but the pattern of writing is not affected by lexical variables such as length, frequency or word class (e.g., Ellis, 1988; Papagno, 1992).

The dissociation between typewriting and speech production, regarding the influence of central processes on peripheral ones is noteworthy. At first glance, it seems counterintuitive that for one type of production behavior differences during central processing cascade to peripheral processing while for another type of production behavior they do not. However, just as argued for the observed differences between speaking and typing with respect to central processing, this could be related to a difference in “proficiency” between both skills. While we engage in speech acts continuously, the amount of typing we perform daily will be much lower for most people. Hence, an interesting (and perhaps even domain-general) question which surfaces from our study is whether the amount of cascading is (among other factors) dependent on the amount of automaticity and attention underlying a particular skill. In this manner, during speech production there may be sufficient “room” to optimize processing through cascading and already engage in motor execution prior to the completion of speech planning. In contrast, for those with less practice at typing, cascading between planning and execution might be more limited because of the higher processing demands on both central processes for retrieving the correct graphemic information and on peripheral factors, such as the frequency of diagraphs or the physical difficulty of the typing movement (two hands vs. one hand; Gentner et al., 1988). It will be very interesting to see in future research whether for more experienced typers (e.g., clerks, scientists, etc.) or for a more frequently used form of typing (text messaging on mobile phone), central processing will demonstrate earlier lexical effects as well as cascading between central and peripheral processes, just as encountered for its spoken counterpart.

In sum, our data demonstrated two marked differences for typewriting compared to speech production: First, lexical access is delayed by some 200 ms for typewriting compared to speaking. Second, while in speech production processing differences arising at the level of planning can affect later execution, in typewriting central and peripheral processes seem relatively independent from each other. These results document that speaking and typing do not just differ with respect to their output modality, but also with respect to the processing dynamics underpinning both types of production behavior. We tentatively suggest that these processing differences might be caused by differences in the amount of automaticity and attention required for performing a speech vs. type act.

### Conflict of interest statement

The authors declare that the research was conducted in the absence of any commercial or financial relationships that could be construed as a potential conflict of interest.

## Footnotes

^1^Besides word frequency, this early ERP effect has been demonstrated to be sensitive to a range of lexical phenomena, such as cognate-status (e.g., Strijkers et al., 2010), semantic interference (e.g., Costa et al., 2010) and response language (e.g., Strijkers et al., 2010, 2013) and therefore traced back to the onset of lexical access when preparing a word for speech (see Strijkers and Costa, 2011).

^2^In the spoken modality, there is convergent evidence showing shorter articulatory durations for high than for low-frequency words (Balota and Chumbley, 1990; Kawamoto et al., 1998; Kello et al., 2000; Damian et al., 2001; Kello, 2004; Gahl, 2008; but see, Wright, 1979; Meyer, 1991; Sadat et al., 2012).

^3^In the 0–60 time-window neither the frequency effect [*F*_(18)_ = 1.12, *p* = 0.30], nor its interaction with region (*F* < 1) were significant.

^4^For typewriting models, such as the *Activation-Trigger-Schemata*, proposed by Rumelhart and Norman (1982), the lack of frequency effects in typing could be explained as a result of the sequential activation of the motor commands of the letters composing a word.

## Appendix

**Table A1:** **Full list of materials**.

**High-frequency words**		**Low-frequency words**	
**Spanish names**	**English translation**	**Spanish names**	**English translation**
Abrigo	Coat	Abeja	Bee
Anillo	Ring	Ancla	Anchor
Armario	Closet	Ardilla	Squirrel
Banco	Bench	Bailarina	Dancer
Bandera	Flag	Ballena	Whale
Barco	Boat	Bate	Bat
Bicicleta	Bike	Bombilla	Light Bulb
Boca	Mouth	Bota	Boat
Botella	Bottle	Bruja	Witch
Caballo	Horse	Canguro	Kangaroo
Cabra	Goat	Cazo	Pot
Cadena	Chin	Cebolla	Onion
Caja	Box	Cebra	Zebra
Calendario	Calendar	Cereza	Cherry
Cama	Bed	Cesta	Basket
Camisa	Shirt	Cigarro	Cigarrette
Campana	Bell	Cocodrilo	Crocodile
Carro	Cart	Cometa	Kite
Cerebro	Brain	Conejo	Rabbit
Corbata	Tie	Cubo	Bucket
Corona	Crown	Cuchara	Spoon
Disco	Record	Dedal	Thimble
Esqueleto	Skeleton	Elefante	Elephant
Falda	Skirt	Faro	Lighthouse
Flor	Flower	Fresa	Strawberry
Gato	Cat	Gallo	Rooster
Globo	Globe	Guante	Glove
Huevo	Egg	Hacha	Axe
Libro	Book	Lazo	Bow
Manzana	Apple	Mariposa	Butterfly
Mesa	Table	Martillo	Hammer
Muñeca	Doll	Moto	Motorbike
Oreja	Ear	Oveja	Sheep
Perro	Dog	Pato	Duck
Piano	Piano	Peine	Comb
Pipa	Pipe	Pera	Pear
Pistola	Gun	Piña	Pinneapple
Plato	Plate	Pinza	Clothespin
Puño	Fist	Pulpo	Octopus
Puro	Cigar	Pimiento	Pepper
Regalo	Present	Rana	Frog
Reloj	Watch	Raqueta	Racket
Silla	Chair	Seta	Mushroom
Sombrero	Hat	Silbato	Whistle
Taza	Mug	Tambor	Drum
Tren	Train	Tigre	Tiger
Tronco	Log	Tomate	Tomato
Zapato	Shoe	Zorro	Fox
